# The Anti-virulence Efficacy of 4-(1,3-Dimethyl-2,3-Dihydro-1H-Benzimidazol-2-yl)Phenol Against Methicillin-Resistant *Staphylococcus aureus*

**DOI:** 10.3389/fmicb.2019.01557

**Published:** 2019-07-17

**Authors:** Nagendran Tharmalingam, Rajamohammed Khader, Beth Burgwyn Fuchs, Eleftherios Mylonakis

**Affiliations:** Department of Medicine, Division of Infectious Diseases, Warren Alpert Medical School of Brown University, Rhode Island Hospital, Providence, RI, United States

**Keywords:** anti-virulent molecules, *agrA*, bacterial virulence, *codY*, methicillin-resistant *Staphylococcus aureus*

## Abstract

Antimicrobial drug discovery against drug-resistant bacteria is an urgent need. Beyond agents with direct antibacterial activity, anti-virulent molecules may also be viable compounds to defend against bacterial pathogenesis. Using a high throughput screen (HTS) that utilized *Caenorhabditis elegans* infected with methicillin-resistant *Staphylococcus aureus* (MRSA) strain of MW2, we identified 4-(1,3-dimethyl-2,3-dihydro-1H-benzimidazol-2-yl)phenol (BIP). Interestingly, BIP had no *in vitro* inhibition activity against MW2, at least up to 64 μg/ml. The lack of direct antimicrobial activity suggests that BIP could inhibit bacterial virulence factors. To explore the possible anti-virulence effect of the identified molecule, we first performed real-time PCR to examine changes in virulence expression. BIP was highly active against MRSA virulence factors at sub-lethal concentrations and down-regulated virulence regulator genes, such as *agrA* and *codY*. However, the benzimidazole derivatives omeprazole and pantoprazole did not down-regulate virulence genes significantly, compared to BIP. Moreover, the BIP-pretreated MW2 cells were more vulnerable to macrophage-mediated killing, as confirmed by intracellular killing and live/dead staining assays, and less efficient in establishing a lethal infection in the invertebrate host *Galleria mellonella* (*p* = 0.0131). We tested the cytotoxicity of BIP against human red blood cells (RBCs), and it did not cause hemolysis at the highest concentration tested (64 μg/ml). Taken together, our findings outline the potential anti-virulence activity of BIP that was identified through a *C. elegans*-based, whole animal based, screen.

## Introduction

Fatal infections from antibiotic-resistant bacteria are predicted to rise and expected to exceed deaths caused by cancer by the year 2050 (de Kraker et al., 2016), therefore putting forth a critical need for novel antibiotics (Zaman et al., 2017). The infection process is a multifaceted process with the interplay between the invading bacteria and host. Most bacterial pathogens rely on virulence factors, inclusive of toxins, adhesins, secreted proteins, secretion systems, lytic enzymes, invasins, etc. to establish an infection (Defoirdt, 2017). Targeting these virulence mechanisms can be a means of thwarting the microbial attack (Wilson et al., 2002).

A possible benefit to developing anti-virulence agents is the potential to reduce antibiotic resistance from overexposure to antibiotic compounds (Hauser et al., 2016). Anti-virulence compounds neither kill nor inhibit bacterial growth, but disable the infection process (Heras et al., 2015; Totsika, 2017). The fundamental principle behind blocking virulence factors is to make the pathogen more vulnerable to host immune responses (Longdon et al., 2015). Currently, anti-virulence compounds present a potentially untapped strategy in controlling or treating bacterial infections.

Virulence factors are already a target exploited by nature to curb microbial infections. Natural products exist that target microbial virulence factors. Examples include garlic, menthol, clove, and black pepper which inhibit enterotoxins, the type III secretion system (T3SS), and biofilm (Maura et al., 2016; Silva et al., 2016). Anti-virulence compounds can also be found among orphaned United States-Food and Drug Administration (FDA)-approved drugs and existing therapeutic agents by engaging in “drug repurposing” (Rampioni et al., 2017). For example, anthelmintic drug niclosamide was found to have a potential “anti-quorum sensing” activity against the Gram-Negative pathogen *Pseudomonas aeruginosa* (Imperi et al., 2013). Recently, the FDA approved a therapy that targets virulence factors using immunoglobulins, while other anti-virulence molecules are in preclinical evaluation (Dickey et al., 2017).

In search of new antimicrobial agents, we performed a high throughput screen (HTS) to find compounds that prolong the survival of *C. elegans* infected with methicillin-resistant *Staphylococcus aureus* (Rajamuthiah et al., 2014; Kim et al., 2018). In this screen, the molecule BIP was identified as a hit. The compound is a benzimidazole derivative linked with phenol. Benzimidazole is a heterocyclic aromatic organic compound consisting of a fusion of benzene and imidazole (Shrivastava et al., 2017) and its derivatives exhibit a diverse biological activity including, anthelminthic, antibacterial, anticancer, anti-inflammatory, etc. (Singh et al., 2012). Because the compound did demonstrate *in vitro* inhibition activity against the MW2, we hypothesized that BIP exhibits anti-virulence activity.

## Materials and Methods

### Compound Information

4-(1,3-Dimethyl-2,3-dihydro-1H-benzimidazol-2-yl)phenol (Chembridge # 5534382) (BIP) was purchased from Chembridge (Chembridge Corporation, San Diego, CA, USA). BIP was prepared by dissolving in DMSO (Sigma Aldrich, St. Louis, MO, USA) at 10 mg/ml and diluting as required for experiments. Hits were identified from the screen based on the Z-score. The Z-score was calculated from the ratio of live versus dead worms after treatment with investigational compounds, and the score is defined as the number of standard deviations (SD) by which the investigational compound is separated from the mean using the formula *Z* = (*x* − *μ*)/*σ*, where *x* is the raw sample score, *μ* is the mean of the population, and *σ* is the standard deviation of the population. Samples with *Z* > 2*σ* were considered as hits (Rajamuthiah et al., 2014). Other approved clinical drugs were purchased from Sigma Aldrich.

### Bacterial and Nematode Strains

All reference and clinical bacterial strains were from our laboratory collection (Table 1). For all experiments, bacteria were grown at 37°C. *S. aureus* (MRSA strain MW2) (clinical strains BF 9 and BF 10) *agr* mutant *S. aureus* RN 4220, *Enterococcus faecium, Klebsiella pneumoniae*, *Acinetobacter baumannii*, *Pseudomonas aeruginosa*, *Enterobacter aerogenes*, and *Candida albicans* were grown in tryptic soy broth (TSB) (BD Biosciences, Franklin Lakes, NJ, USA). The *C. elegans glp-4(bn2);sek-1(km4)* strain was maintained at 15°C on a lawn of *Escherichia coli* strain HB101 on 10-cm plates, as previously described (Rajamuthiah et al., 2014). The *glp-4(bn2)* mutation renders the strain unable to produce progeny at 25°C (Beanan and Strome, 1992), and the *sek-1(km4)* mutation increases sensitivity to several pathogens (Tanaka-Hino et al., 2002), thereby reducing assay time.

**Table 1:** Antimicrobial activity of BIP against the major pathogens.

Pathogen	MIC (μg/ml)
*Staphylococcus aureus MW2*	>64
*Enterococcus faecium* ATCC E007	>64
*Acinetobacter baumanii* ATCC 17978	>64
*Enterobacter aerogens* EAE 2625	>64
*Klebsiella pneumoniae* ATCC 77326	>64
*Pseudomonas aeruginosa* PA14	>64
*Candida albicans* SC 5314	>64

### Antimicrobial Susceptibility Testing

*In vitro,* antibiotic susceptibility assays were carried out using a broth microdilution assay using Müller-Hinton broth (BD Biosciences) in 96-well plates (BD Biosciences) with a total assay volume of 100 μl (Wikler, 2006). Two-fold serial dilutions of BIP were prepared over the concentration range 0.01 – 64 μg/ml. The initial bacterial inoculum was adjusted to OD_600_ = 0.06 and assays were incubated at 35°C for 18 h. To determine the minimum inhibitory concentration (MIC), OD_600_ was measured and visual inspection was made after incubation, and the lowest concentration of test compound that suppressed bacterial growth was reported as the MIC. The choice of medium for *C. albicans* was RPMI and 10^3^–2.5 × 10^3^ of initial inoculum was used to determine the MIC. The assays were carried out in triplicate.

### Time to Kill Assays

The strain MW2 was used to probe the killing effects of BIP, as previously described (Rajamuthiah et al., 2015). The assays were carried out in 10-ml tubes (BD Biosciences) in duplicate. Briefly, log-phase cultures of MW2 were diluted in fresh TSB to a density of 10^8^ cells/ml. BIP was added at sub-lethal concentrations (64, 32 μg/ml), and the tubes were incubated at 37°C with agitation. At periodic intervals, aliquots from each tube were serially diluted in TSB and plated onto tryptic soy agar (TSA, BD Biosciences). CFU was enumerated after overnight incubation at 37°C (Tharmalingam et al., 2017). Gentamicin at 0.5 μg/ml (MIC: 1.0 μg/ml) was used as a control agent.

An independent experiment was carried out to test the hypothesis of post-antibiotic effect. The MW2 cells were treated with BIP (64 μg/ml) for 4 h, and the cells were washed with sterile PBS. The washed cells were suspended in fresh MHB (0.05 OD_600_) and incubated for 2 h with agitation. After 2 h, the total viable count was enumerated as described earlier in this section.

### Human Red Blood Cell Hemolysis

Human erythrocytes (Rockland Immunochemicals, Limerick, PA, USA) were used to test the hemolytic properties of BIP, as described previously (Isnansetyo and Kamei, 2003). BIP was serially diluted in PBS, and an equal volume of 4% RBCs were added in 96-well plates. After incubating at 37°C for 1 h, the plates were centrifuged at 500 × *g* for 5 min, and an aliquot of 100 μl of the supernatant from each well was transferred to a second 96-well plate. Triton-X (0.0025–1.0%) was used as a positive control. Visual inspection and absorbance at 540 nm were used to measure hemolysis.

### Real-Time Quantitative PCR Assays

An overnight culture of MW2 was grown in TSB to evaluate the effects of BIP on bacterial gene expression. The culture was harvested when the OD_600_ had reached 0.4. The cells were washed with PBS and exposed to the sub-lethal concentration of BIP (32 or 64 μg/ml), vancomycin (0.5 μg/ml), or DMSO for 4 h. RNA was prepared using RNeasy mini kit (Qiagen, Hilden, Germany) according to the manufacturer’s instructions. The Verso cDNA synthesis kit (Thermo Fisher Scientific, MA, USA) was used for cDNA synthesis. Real-time quantitative polymerase chain reaction (RT-qPCR) was performed as described in the manufacturer’s instructions (Bio-Rad, CA, USA) using the primers reported by Liu et al. (2018) and the iCycler iQ real-time detection system (Bio-Rad). No-RT control was included as a negative control. According to Mitchell et al. (2011), the relative expression ratios were calculated as follows: n-fold expression = 2^‑ΔΔCt^, ΔΔCt = ΔCt (drug-treated)/ΔCt (untreated), where ΔCt represents the difference between the cycle threshold (Ct) of the gene studied and the Ct of housekeeping 16S rRNA gene (internal control). Primer sequences used in this study were: 5-GGGACCCGCACAAGCGGTGG-3 and 5-GGGTTGCGCTCGTTGCGGGA-3 (Atshan et al., 2013). Statistical significance was calculated using Student’s *t*-test.

### Intracellular Killing Assays

RAW 264.7 macrophages were used to evaluate the intracellular killing of BIP-pretreated *S. aureus*. Assays were performed in triplicate as described by Schmitt et al. (2013). Briefly, macrophages were cultured and maintained as described previously (Jayamani et al., 2017). Macrophages (5 *×* 10^5^) were seeded in 24-well plates 24 h before infection. The multiplicity of infection (MOI) of 25 (i.e., 25 bacterial cells per macrophage) was used for the assay in which BIP-pretreated MW2, untreated MW2 alone, or *agr* mutant *S. aureus* RN4220 cells were added to macrophage cultures for 2 h to interrogate phagocytosis. After the incubation period, planktonic bacteria were removed, and DMEM containing 200 μg/ml gentamicin was added for 2 h to inhibit/kill remaining extracellular bacteria. The cells were incubated under 5% CO_2_ for 20 h with gentamicin (1X MIC, 1 μg/ml) to prevent the multiplication of bacterial cells released from burst macrophages. After incubation, the macrophages were lysed by adding SDS to a final concentration of 0.02% (i.e., lysing only macrophages and not ingested bacteria). Cell lysates were diluted serially and the CFUs were enumerated by plating on TSA plates (Tharmalingam et al., 2017).

### Live/Dead Staining Assay

The macrophage phagocytosis assay was performed as discussed in the intracellular killing assay above, except cells were not lysed. Instead, after 20 h of incubation, the cells were washed twice with 0.1 M 3-(N-morpholino) propanesulfonic acid (MOPS), pH 7.2, containing 1 mM MgCl_2_ (MOPS/MgCl_2_). Then, the cells were stained with live/dead staining solution (5 μM SYTO9, 30 μM propidium iodide and 0.1% saponin in MOPS/MgCl_2_) and incubated for 15 min in the dark. The cells were then rinsed with MOPS/MgCl_2_ and observed using a Nikon C1si 141 confocal microscope (Nikon, Melville, NY, USA) with 488- and 561-nm diode lasers and images were captured (Zheng et al., 2017b).

### Checkerboard Assay

Compounds were arrayed in serial concentrations, vertically for one molecule and horizontally for the other molecule in 96-well plate. The bacterial inoculation and measurement of growth were carried out as described earlier in the antimicrobial susceptibility assay. The synergy was measured by calculating the fractional inhibitory concentration index (FICI) using the formula FICI = (*A*/MIC*A*) + (*B*/MIC*B*), whereby MIC*A* and MIC*B* are the MIC’s individual molecules, and (*A*) and (*B*) are the lowest concentration of the molecule in combination with another molecule that inhibits bacterial growth. An FICI of <0.5 indicated synergism and scores between 0.51 and 1.0 suggest a partial synergy between the compounds being tested (Odds, 2003).

### *Galleria mellonella* Methicillin-Resistant *Staphylococcus aureus* Infection Assay

Sixteen *G. mellonella* larvae (Vanderhorst Wholesale, St. Mary’s, OH, USA), which were randomly selected and weigh between 300 and 350 mg, were used for each group in the experiment interrogated group (Gibreel and Upton, 2013). Overnight, MW2 cells were diluted at 1:2,000 in a cation-adjusted MHB in the presence of a sub-lethal concentration of BIP (64 μg/ml) or vancomycin (0.5 μg/ml). After incubation with agitation for 12 h, the cells were washed and suspended in PBS at an optical density of absorbance 600 (OD_600_) of 0.3. A 10-μl (2 × 10^6^ cells/ml) inoculum was injected into the last left proleg using a Hamilton syringe and incubated at 37°C. Five test groups were included, and the same dose of bacteria was injected into the corresponding infection groups: (1) PBS alone (no bacteria control), (2) BIP-pretreated MW2 cells, (3) vancomycin-pretreated MW2 cells, (4) DMSO-pretreated MW2 cells, and (5) no injection and no bacteria (quality control). *G. mellonella* survival was assessed up to 120 h, with larvae considered dead if unresponsive to touch using sterile tips. Killing curves and differences in survival were analyzed by the Kaplan–Meier method using GraphPad Prism version 6.04 (GraphPad Software, La Jolla, CA, USA). All of the statistical analysis was carried out using the same program, and *p* < 0.05 was considered significant.

## Results and Discussion

### Antibacterial Susceptibility

**Figure 1:**
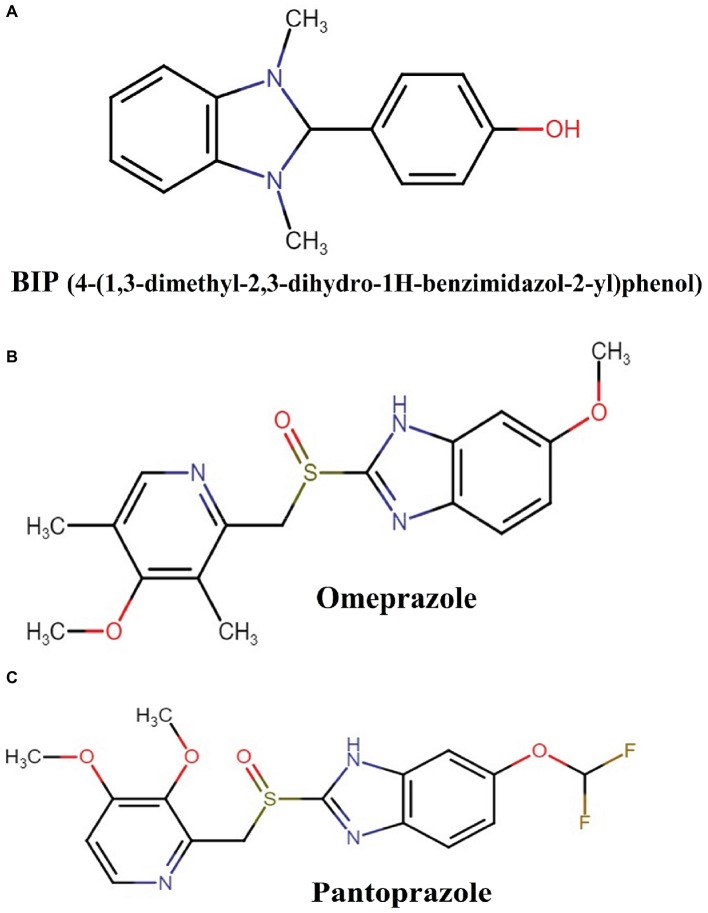
Chemical structures of: **(A)** BIP (4-(1,3-dimethyl-2,3-dihydro-1H-benzimidazol-2-yl)phenol), **(B)** omeprazole, and **(C)** pantoprazole.

We previously reported several direct antibacterial molecules that were discovered using a whole organism *C. elegans*-based HTS (Rajamuthiah et al., 2014, 2015; Gwisai et al., 2017; Jayamani et al., 2017; Tharmalingam et al., 2017, 2018a,b; Zheng et al., 2017a). In this work, we report a molecule 4-(1,3-dimethyl-2,3-dihydro-1H-benzimidazol-2-yl)phenol (BIP) (Figure 1) that produced an average Z-score of 9.61 and rescued the nematode *C. elegans* from MW2 infection. The benzimidazole derivatives have potent antibacterial activity against Gram-positive (Göker et al., 2005; Tunçbilek et al., 2009; Karataş et al., 2012) and certain Gram-negative bacteria (Picconi et al., 2017; Vashist et al., 2018). Also, studies have reported that the benzimidazole derivatives exhibit an anti-biofilm and anti-virulence role (Kim et al., 2009; Sambanthamoorthy et al., 2011; Kong et al., 2018). Interestingly, although BIP was identified as a hit from the screen, the broth microdilution assay revealed that BIP did not inhibit the growth of *S. aureus* or any other organisms tested (Table 1). The seemingly contradictory results from HTS and broth-microdilution assay generated the hypothesis that the molecule may prolong host survival by either inhibiting bacterial virulence or modulating host immunity. We also tested the antibacterial activity of the benzimidazole derivatives omeprazole and pantoprazole. As previously reported (Sjöström et al., 1996; Ni et al., 2014), neither compound inhibited MW2 growth at the highest concentration tested (64 μg/ml).

### Exploring the Role of 4-(1,3-Dimethyl-2,3-Dihydro-1H-Benzimidazol-2-yl)Phenol on MW2 Growth

**Figure 2:**
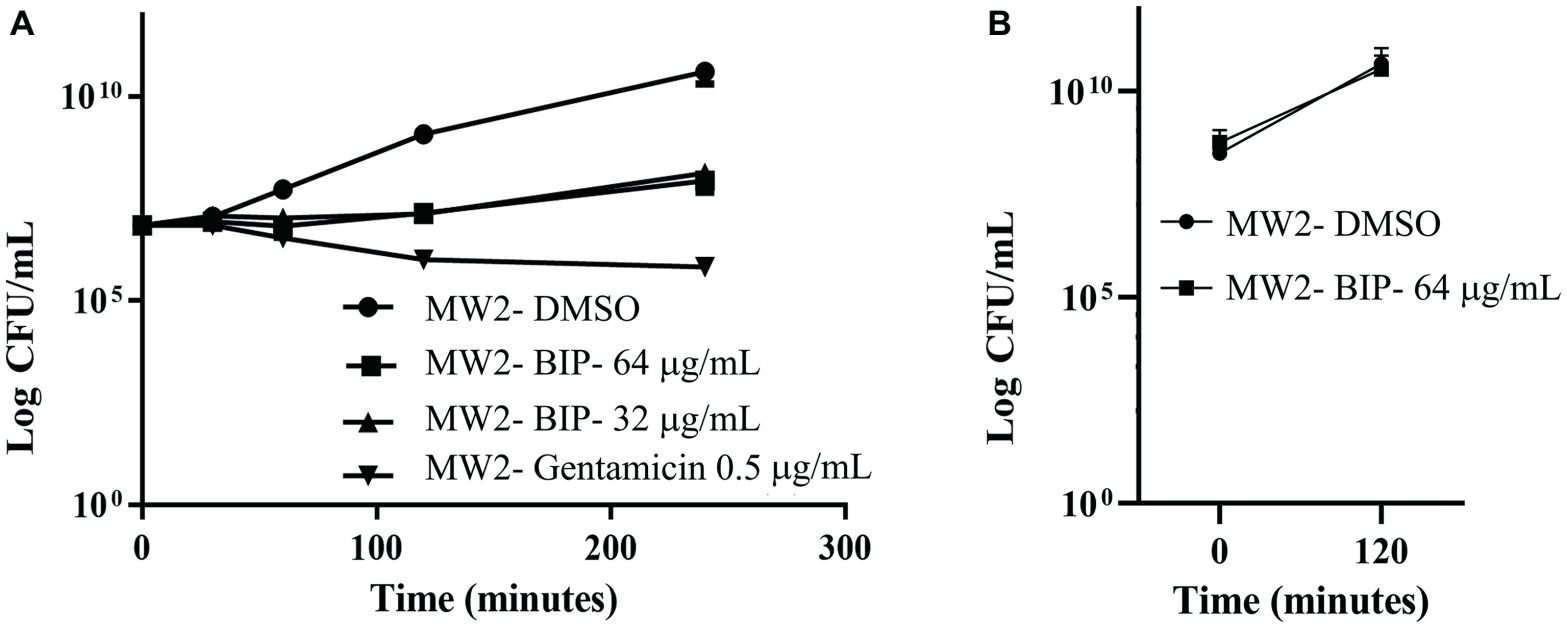
Killing kinetics. Growth curves were generated using *S. aureus* cells. **(A)** After BIP treatment, MW2 cells continued to grow, although at a slower rate than cells exposed to DMSO. **(B)** After BIP was removed by washing, the MW2 cells exhibited normal growth comparable to DMSO pre-treated cells. Data represent the mean ± SD (*n* = 3).

First, we aimed to evaluate whether BIP can decrease the bacterial cell numbers at the concentration of 32 or 64 μg/ml from an initial inoculum. The killing kinetics assay determined that the total viable bacterial count was about 5-logCFU at 0 h. After 4 h, the total viable count of BIP-pretreated cells was about 6-logCFU (Figure 2A). The DMSO-treated MW2 cells at the later time point were measured at 10-logCFU. The data suggest that BIP treatment resulted in slower bacterial growth compared to DMSO treatment but did not exert either bacteriostatic or bactericidal activity. As a positive control, we included the antibacterial agent gentamicin at 0.5 MIC (0.5 μg/ml). Gentamicin decreased the initial inoculum by 1-logCFU during the same time period (Figure 2A). In addition, a post-antibiotic effect assay determined that the bacterial cells grew normally compared to DMSO control after the cells were washed post drug treatment (Figure 2B). Taken together, we found that BIP did not decrease overall cell number from an initial inoculum and BIP-pretreated cells grew normally upon drug removal indicating that the effect of the compound is related to altered growth dynamic and not viability.

### Toxicity of 4-(1,3-Dimethyl-2,3-Dihydro-1H-Benzimidazol-2-yl)Phenol on Human Red Blood Cellss

**Figure 3:**
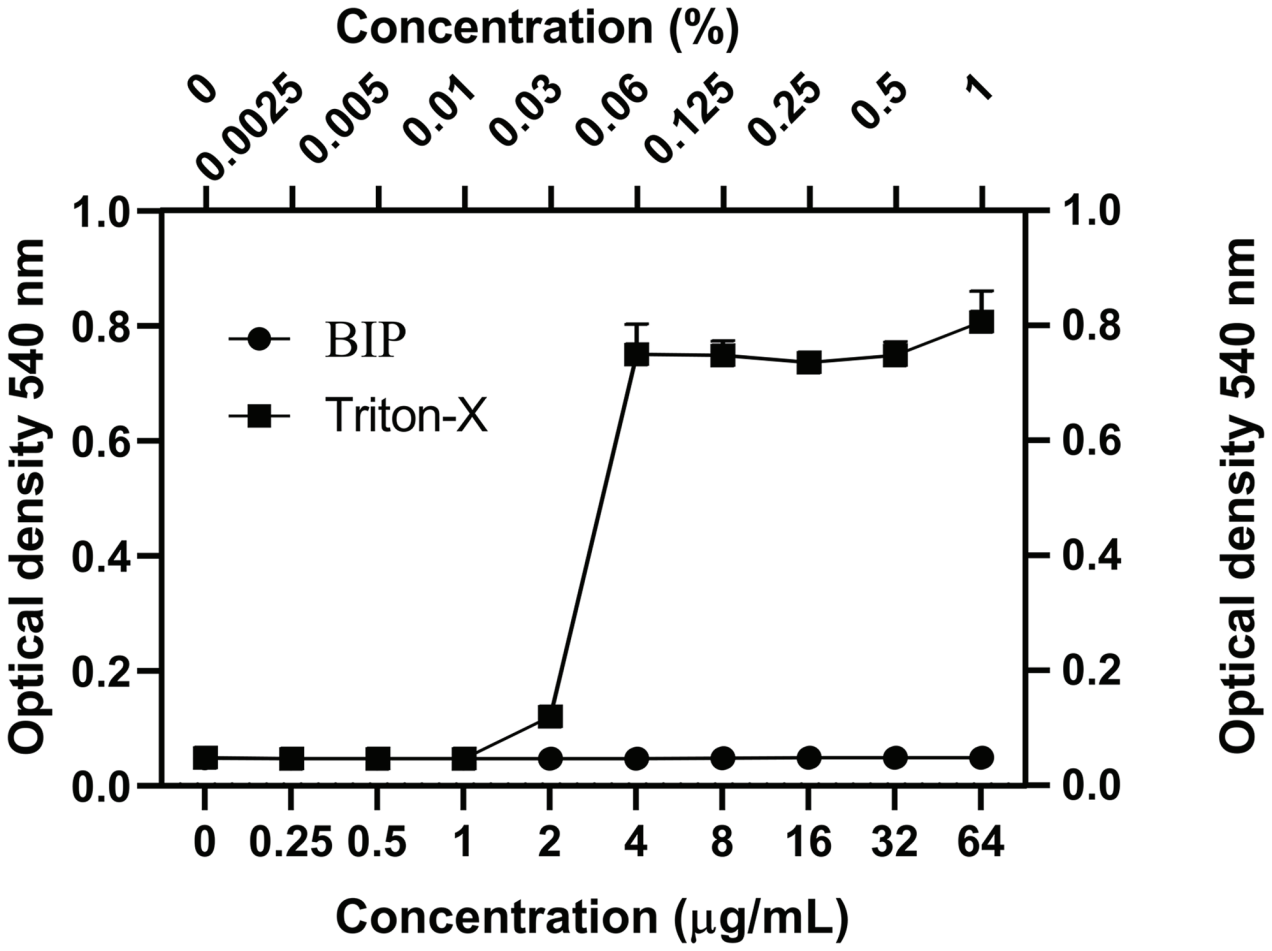
Cytotoxicity assay. Toxicity of BIP was tested using human red blood cells. BIP concentrations up to 64 μg/ml did not cause hemolysis. In this study, 0.0025–1% Triton-X was used as a positive control. Data represent the mean ± SD (*n* = 3).

We tested the hemolysis potential of BIP with human RBCs and found that the BIP treatment did not cause hemolysis up to 64 μg/ml (Figure 3). We used 0.0025–1.0% of Triton-X as a positive control. The lack of detectable RBC cytotoxicity was confirmed in this assay.

### Exploring the Anti-virulence Role of 4-(1,3-Dimethyl-2,3-Dihydro-1H-Benzimidazol-2-yl)Phenol

**Figure 4:**
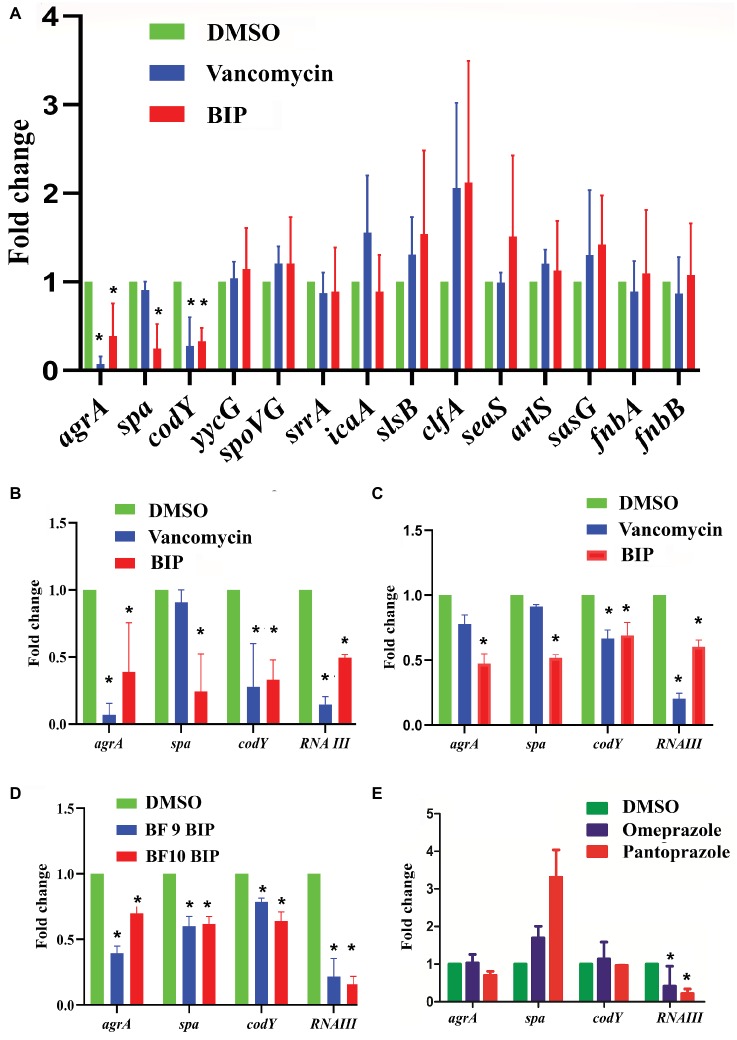
Effect of BIP on virulence genes. *S. aureus* MW2 cells were treated with BIP (64 μg/ml) for 4 h. RNA was isolated and used as a template for cDNA synthesis and was analyzed using real-time PCR. **(A)** Exposure to BIP was associated with down-regulation of major virulence regulator genes, including *agrA*, *spa,* and *codY*. **(B,C)** The expression of virulence genes *agrA, spa, codY, RNA III* was examined during the 4- and 24-h treatments of BIP, respectively. Drug treatment down-regulated *agrA, spa, codY,* and *RNA III* expression. **(D)** Two clinical strains of *S. aureus* (BF 9, BF 10) were also evaluated for changes to *agrA, spa, codY*, and *RNA III* expression. **(E)** MW2 cells were exposed with the benzimidazole derivatives omeprazole and pantoprazole (64 μg/ml) for 4 h. Data represent the mean ± SD (*n* = 3). ^*^p < 0.05, Student’s *t*-test comparing DMSO control.

We examined a panel of known virulence genes (*agrA, spa, codY, yycG, spoVG, srrA, icaA, slsB, clfA, saeS, arlS, sasG, fnbA, fnbB*) to evaluate if they were affected by BIP. Since RNA III is known to control several virulence factors, we also included it in the panel of examined genes. After exposing bacteria to BIP, we monitored the expression of MW2 virulence genes through a real-time PCR assay. The presence of the BIP down-regulated virulence genes such as accessory gene regulator A (*agrA*), staphylococcal protein A (*spa*), and GTP-sensing transcriptional pleiotropic repressor (*codY*) with statistical significance (Figure 4A). Virulence gene expression after the BIP treatment was examined in reference and non-reference strains of *S. aureus*, including two clinical isolates in order to evaluate the effect of conservation. Interestingly, BIP induced down-regulation of *agrA*, *spa*, *codY,* and *RNA III* genes in all the isolates tested, (Figure 4D) demonstrated that the effect is not a strain-specific. To exclude the possibility of a time-dependent effect of BIP treatment, we examined the prolonged treatment (24 h) of BIP and observed a down-regulation of *agrA*, *spa*, and *codY* (Figures 4B,C) in MW2. Benzimidazole derivatives are majorly known for their antimicrobial action (Singh et al., 2012). However, few studies show that these compounds target virulence determinants by inhibiting multiple adaptational response (MAR) transcription factor in various pathogens without targeting bacterial growth (Kim et al., 2009; Grier et al., 2010). These previous studies support our findings that the BIP down-regulates bacterial virulence without targeting bacterial growth *in vitro*.

In *S. aureus*, the *agr* system plays a central control in the regulation of virulence gene expression and is involved in the production of multiple virulence factors (Tan et al., 2018). Also, *agrA* is a response regulator of the *agr* system, and it binds to the recognition site in RNA III and RNA II (Tan et al., 2018). RNA III controls several virulence factors, including the *spa* gene (Huntzinger et al., 2005), supporting our finding that down-regulation of *agrA* by BIP is coupled with down-regulation of the *spa* gene. Shrestha et al. reported that the benzimidazole derivative (ABC-1) down-regulated *spa* primarily, but not *fnbA*, *fnbB*, *clfA*, and *clfB* (Shrestha et al., 2016) supports our study with a similar kind of observation. Exposure to BIP also caused reduced expression of *codY* that produces a global regulator protein known to repress *agrBDCA* and RNA III transcription (Roux et al., 2014). By comparison, vancomycin treatment caused down-regulation of *agrA* and *codY* (Figure 4A) but not *spa*, demonstrating that the sub-MIC level of vancomycin exerted limited influences on *S. aureus* virulence genes expression (Cázares-Domínguez et al., 2015; Hodille et al., 2017). In addition, we tested the anti-virulence activity of the benzimidazole derivatives omeprazole and pantoprazole (Kromer, 1995). Omeprazole at 64 μg/ml did not down-regulate the expression of the major staphylococcal virulence genes *agrA, spa,* and *codY* (Figure 4E). Pantoprazole at 64 μg/ml down-regulated the gene expression of *RNA III* (Figure 4E). These results, along with published reports, indicate that some benzimidazole derivatives down-regulate bacterial virulence (Kim et al., 2009; Sambanthamoorthy et al., 2011; Kong et al., 2018), while other benzimidazole derivatives, such omeprazole, and pantoprazole, do not appear to influence staphylococcal virulence. The two approved drugs, omeprazole and pantoprazole, are proton pump inhibitors (PPIs) and reduce the gastric pH (Axon and Moayyedi, 1996). Structural activity relationship (SAR) studies demonstrated that the omeprazole and methoxy or fluorine-substituted analogs affect urease activity (Kühler et al., 1995). Vidaillac et al. demonstrated that the addition of one methoxy group in the benzimidazole nucleus resulted in improved activity (Vidaillac et al., 2007).

### Macrophage Assay

O’Keeffe et al. reported that the *agr* gene is associated with autophagosome protection of bacterial cells that leads to intracellular survival within phagocytes (O’Keeffe et al., 2015). As shown above, BIP causes down-regulation of *agr* and we investigated whether exposure to BIP affects the susceptibility of MRSA cells during phagocytosis by mouse macrophage cells. In this macrophage assay, MW2 cells were treated with BIP for 4 h, before being introduced into wells with RAW 264.7 cells. Untreated cells and the *agr* mutant strain *S. aureus* RN 4220 were included as controls. The *agrA* mutant strain RN4220 was used as a control in order to contrast the down-regulation of *agrA* in BIP-treated MW2 cells. After 20 h of incubation, live bacterial cells inside the macrophages were enumerated by total viable count and bacterial live/dead stain.

**Figure 5:**
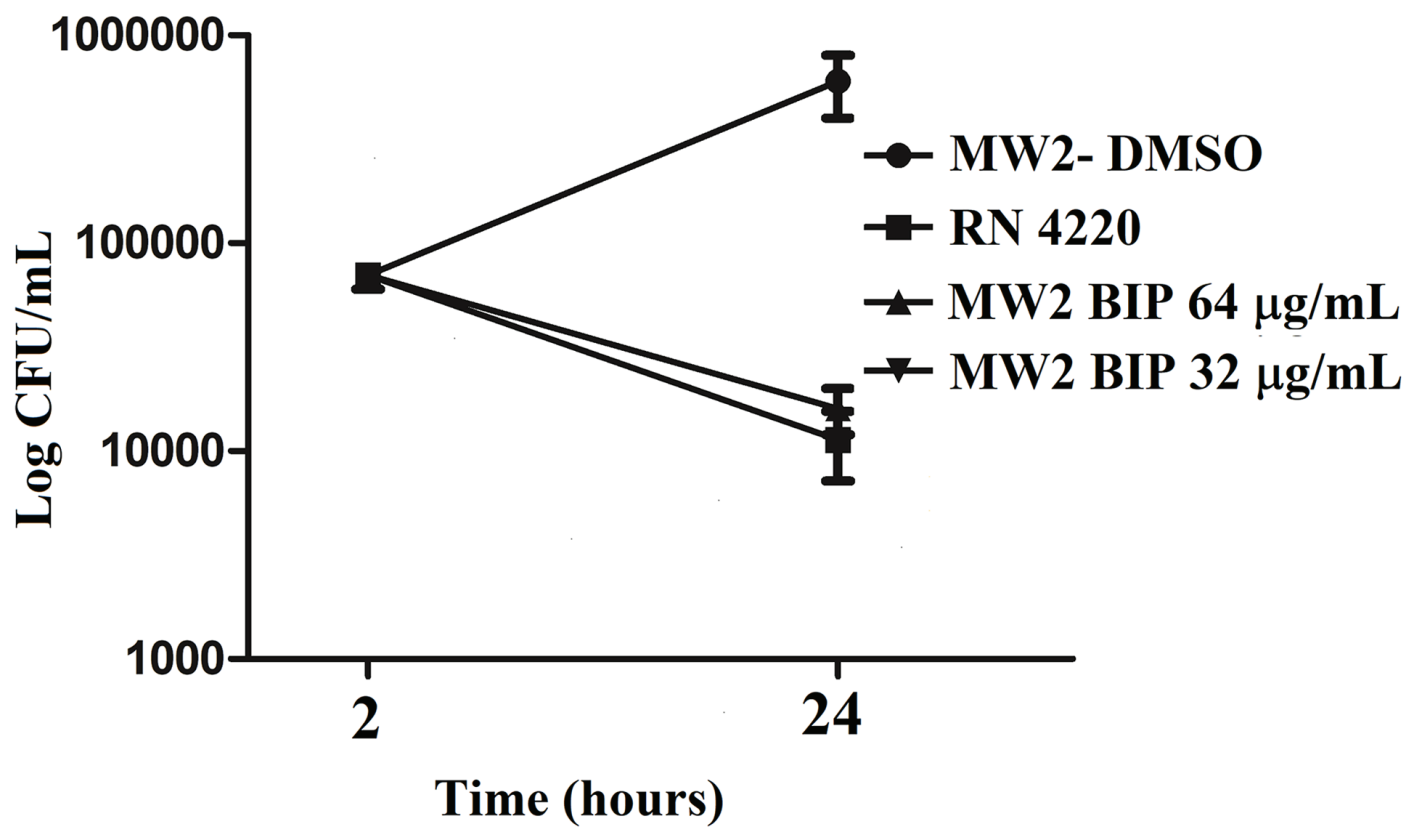
The effect of BIP in the killing of *S. aureus* MW2 cells by mouse macrophages. Raw 264.7 cells were co-incubated with BIP-pretreated MW2 cells, and the viable bacterial cells were enumerated after lysis release. Mouse macrophages killed the BIP-pretreated cells, while DMSO-pretreated cells survived and increased to 1.5-logCFU from the initial inoculation. The *agr* mutant strain *S. aureus* RN4220 was used as a positive control.

We found that the DMSO-pretreated MW2 cells survived and grew inside the macrophages. After 20 h of incubation, the 1.5-logCFU of DMSO-pretreated cells increased from the initially phagocytosed MW2 cells (Figure 5). However, BIP-pretreated cells and *agr* mutant *S. aureus* RN4220 were killed by macrophages, resulting in a 1-logCFU decrease from the initially phagocytosed MW2 cells inside the macrophages (Figure 5). Kong et al. reported that the new benzimidazole derivative (UM-C162) exhibits a strong anti-virulence role by down-regulating the major biofilm forming genes, including the genes responsible for intracellular multiplications (*clpB, clpC, ctsR*) (Kong et al., 2018). Hence, targeting genes responsible for intracellular bacterial survival directly or indirectly results in hindered intracellular multiplication, supporting our observation that the role of BIP on *agrA* down-regulation might contribute in making bacteria more prone to macrophage-mediated killing.

**Figure 6:**
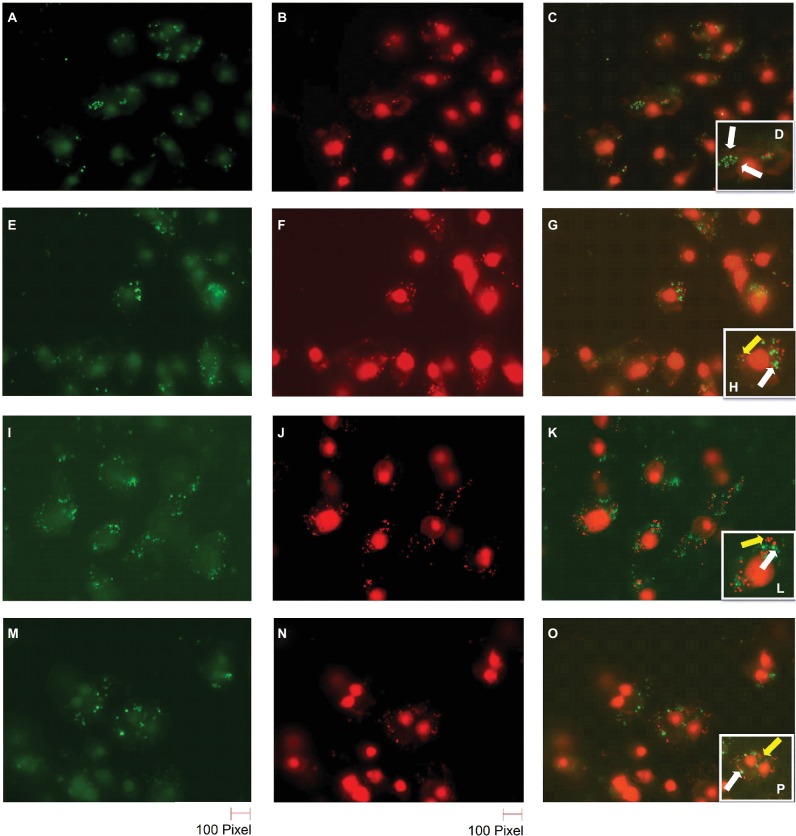
Live/dead staining of BIP-pretreated *S. aureus* MW2 cells within macrophages. Raw 264.7 cells were allowed to phagocytose MW2 cells that were pretreated with either DMSO or BIP. After 20 h, the cells were washed and stained with a bacterial live/dead stain. The fluorescence figures were imaged using two different channels. Live cells are depicted with green fluorescent images **(A,E,I,M)**, and dead cells are represented in red fluorescent images **(B,F,J,N)** (magnification 65×). Images taken in two different channels were merged **(C,G,K,O)** and enlarged images are presented in the panel **(D,H,L,P)**. Live bacterial cells were marked with a white arrow, and dead bacterial cells were marked with a yellow arrow. DMSO-treated bacterial cells **(A–D)** were stained green, while BIP-pretreated bacterial cells (**E–H** – 64 μg/ml and **I–L** – 32 μg/ml) were stained red suggesting that pretreatment with BIP made cells susceptible to macrophage killing. The *agr* mutant strain *S. aureus* RN4220 **(M–P)** was used as a positive control.

*S. aureus* cells can survive inside macrophages (Fraunholz and Sinha, 2012). In order to confirm the reduction of BIP-pretreated bacterial cells and the greater susceptibility of the *agr* mutant strain, we performed bacterial live/dead stain (green/red color) to differentiate viable versus dead cells inside the macrophages (Figures 6A–D). We found that the DMSO-pretreated MW2 cells fluoresced green (Figures 6A–D), indicating bacterial survival, and BIP-pretreated bacterial cells (64 μg/ml, Figures 6E–H; 32 μg/ml, Figures 6I–L) stained red, indicating death. These studies confirmed our findings observed through CFU-based data. We also used the *S. aureus* RN 4220 (an *agrA* mutant strain) to confirm the hypothesis that down-regulation of *agrA* makes bacteria more susceptible to macrophage-mediated killing (O’Keeffe et al., 2015). Indeed, the strain RN 4220 was more susceptible to macrophage-mediated killing (Figures 6M–P), than a wild strain of MW2.

### 4-(1,3-Dimethyl-2,3-Dihydro-1H-Benzimidazol-2-yl)Phenol Potentiates Antibiotic Activity

**Figure 7:**
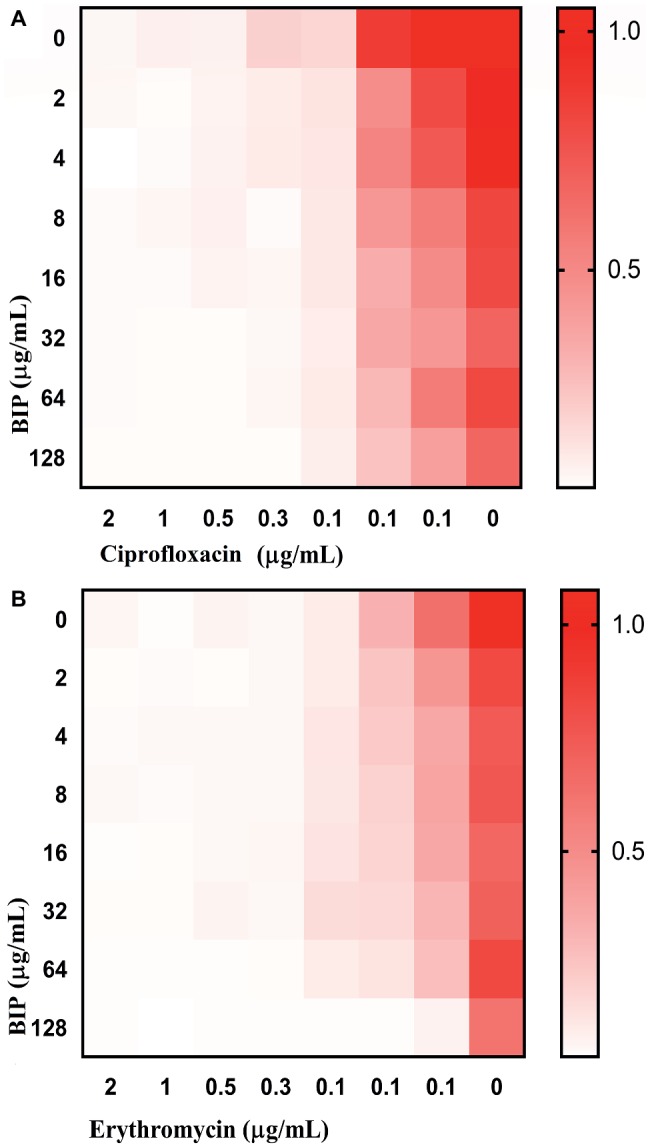
Enhanced activity of clinical antibacterial agents in association with BIP. In the checkerboard assay, BIP demonstrated partial synergistic activity (FICI: 0.75) with **(A)** ciprofloxacin or **(B)** erythromycin. The representative figure is from triplicate repeats. The white color indicates no growth, and red color indicates a gradual increase in growth.

Anti-virulence molecules work synergistically with conventional antibiotics to impair bacterial cells (van Tilburg Bernardes et al., 2017). For example, Abraham et al. reported that the tick anti-virulence protein IAFGP demonstrated a synergistic role with conventional antibiotics (Abraham et al., 2017). Hence, we evaluated if BIP was synergistic with other conventional antibiotics, testing a representative from the most common antibiotics, including β-lactam, fluoroquinolones, peptide antibiotics, tetracyclines, macrolides, and aminoglycosides. In a checkerboard assay, BIP enhanced the antibiotic activity of ciprofloxacin and erythromycin (Figure 7) with a FICI of 0.75, a value that indicates partial synergy (Martin et al., 1996). Fractional Inhibitory Concentration Index (FICI) = (*A*/MIC*A*) + (*B*/MIC*B*), where MIC*A* and MIC*B* are the MIC’s individual molecules, and (*A*) and (*B*) are the lowest concentration of the molecule in combination with another molecule that inhibits bacterial growth. An FICI of <0.5 indicates synergism, while scores between 0.51 and 1.0 suggest a partial synergy between the compounds being tested (Odds, 2003).

### Insect Survival

**Figure 8:**
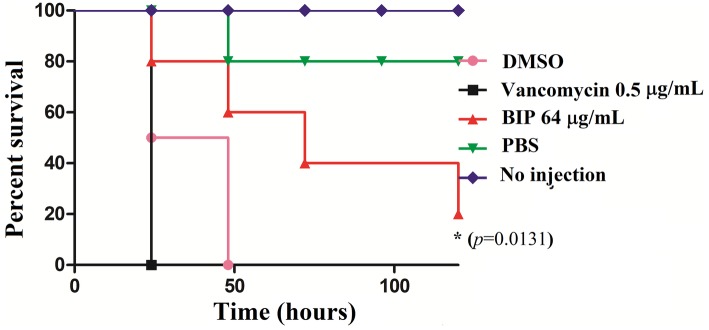
*In vivo* killing efficacy of BIP-pretreated *S. aureus* MW2 cells in the *G. mellonella* model. MW2 cells were pretreated with BIP or DMSO for 12 h prior to injection to *G. mellonella* larva (*n* = 16). After 24 h, the group of larvae that received the BIP-pretreated cells survived longer than the group that received the DMSO-pretreated bacterial cells (*p* = 0.0131). As a control, the group of larvae that received vancomycin-pretreated MW2 cells also killed the larvae within 24 h from incubation. The figure is a representative graph from three independent replicates.

*G*. *mellonella* is used widely to investigate various pathogens (Gibreel and Upton, 2013; Giannouli et al., 2014; Yang et al., 2017), and test new investigational compounds (Desbois and Coote, 2012). Given that BIP-pretreated cells were more susceptible to macrophage killing, we hypothesized that they would be less efficient in killing an infected host. We injected DMSO-treated MW2 cells versus BIP-pretreated MW2 cells into *G. mellonella* larvae. After 24 h, all larvae injected with DMSO-treated MW2 cells were dead (100%). However, at the same time point, 80% of BIP-pretreated (64 μg/ml) MW2 injected larvae survived (*p* = 0.131; Figure 8). Starkey et al. reported that the benzamide-benzimidazole phenoxy substituted benzamide ring that contains a benzimidazole moiety rescue mice from *P. aeruginosa* infection by reducing its acute virulence without reducing bacterial CFU (Starkey et al., 2014). Garrity et al. demonstrated that non-antibacterial LcrF inhibitors protected mice significantly from lethal *Yersinia pseudotuberculosis* lung infection (Garrity-Ryan et al., 2010). These reports support our insect survival data and, taken together, these observations suggest that the anti-virulence molecules harboring the benzimidazole moiety can prevent the invertebrate/vertebrate animals from lethal bacterial infection. Vancomycin is known to prolong the survival of *S. aureus*-infected *G. mellonella* (Tharmalingam et al., 2017). However, in the presented assay, a sub-MIC concentration was provided to *G. mellonella* as a positive control to induce a moderate inhibition of bacteria within the host. The provision of vancomycin at sub-MIC levels was shown to reduce expression of *agrA, spa,* and *codY* as was also seen with BIP treatment (Figure 4). However, a distinction between vancomycin and BIP treatment was noted with *G*. *mellonella* survival in which BIP prolonged survival, whereas vancomycin treatment did not.

The finding suggests that exposure of MW2 cells to BIP results in down-regulation of major virulence factors, such as *agrA, spa, codY*. Moreover, the MW2 cells become more vulnerable to phagocytosis-mediated bacterial killing and, in turn, less virulent in a model host. Based on our findings, we conclude that BIP is an anti-virulence candidate compound that deserves further study. The current data lead us to believe that BIP acts in a capacity to inhibit *S. aureus* virulence factors. The fact that BIP treatment contributes to staphylococcal killing by macrophages and results in prolonged survival of the infected alternative host suggests that the compound might act through host immune modulation (which we do not rule out as a contributing influence) and/or inhibition of virulence attributes needed during the infection process. Apart from an anti-virulence role, BIP might modulate host immunity, but this is beyond the scope of this report. Nevertheless, the effects of benzimidazole derivatives to host immunity are noted in the literature. Wildenberg et al. reported that six benzimidazole derivatives induced regulatory macrophages (Wildenberg et al., 2017). Rickard et al. reported that the benzimidazole diamide GSK 669 inhibits NOD2, an intracellular pattern recognition receptor involved in the induction of pro-inflammatory cytokines (Rickard et al., 2013). Moreover, the benzimidazole derivatives are reported to have an analgesic action (Srivastava et al., 2012). In another study, the benzimidazole derivatives are a potential candidate for the management of opioid-induced paradoxical pain (Idris et al., 2019). As demonstrated by Alpan et al., BIP compounds can also bind with mammalian DNA (Alpan et al., 2007). Further SAR studies demonstrated that the methylated BIP (5-methyl-4-(1H-benzimidazole-2-yl)phenol) derivative exerts increased topo-isomerase I inhibition (Alpan et al., 2007). Interestingly, Gul et al. reported that the benzimidazole phenol derivative (4-(1H-benzimidazol-2-yl)phenol) inhibits human carbonic anhydrase (hCA) activity and addition of more phenol side group decreases the level of inhibition (Gul et al., 2016). Taken together, these reports suggest that the benzimidazole compounds potentially have multiple targets (Singh et al., 2012). In drug discovery, we often find that compounds do not have a singular effect or target; indeed, effects can be multifaceted. This withstanding, our current research does provide strong evidence that BIP inhibits known virulence genes. Future work is needed to determine the specific mechanisms that convey the anti-virulence effect.

## Author Contributions

NT and EM conceived the idea and designed the study. NT designed the project, performed the experiments, and analyzed the data. RK performed the RT-qPCR and insect survival assays. NT drafted the manuscript with revisions and final approval by BF and EM. EM supervised the project.

### Conflict of Interest Statement

The authors declare that the research was conducted in the absence of any commercial or financial relationships that could be construed as a potential conflict of interest.
